# Iron limitation-induced modulation of transcription in *Chlamydia trachomatis*

**DOI:** 10.1038/s41598-026-48043-y

**Published:** 2026-04-27

**Authors:** Daniel Rodriguez Rozo, Simone E. Adams, Carole Kebbi-Beghdadi, Trestan Pillonel, Sébastien Aeby, Gilbert Greub

**Affiliations:** 1 Institute of Microbiology, Lausanne University Hospital and Lausanne University, Lausanne, Switzerland; 2 Service of Infectious Diseases, Lausanne University Hospital and Lausanne University, Lausanne, Switzerland

**Keywords:** *Chlamydiota*, *Chlamydia trachomatis*, *Waddlia chondrophila*, RNA-seq, Transcriptomics, Aberrant bodies, Persistence, Iron deprivation, Stress response, Reversion, Two-component system, Diseases, Genetics, Microbiology, Molecular biology

## Abstract

*Chlamydia trachomatis* (*C. trachomatis*) is an obligate intracellular bacterial pathogen responsible for trachoma and sexually transmitted infections. Like other *Chlamydiota* members, such as *Waddlia chondrophila*, *C. trachomatis* undergoes a biphasic developmental cycle alternating between infectious elementary bodies (EBs) and replicative reticulate bodies (RBs). Under stressful conditions, RBs differentiate into non-dividing aberrant bodies (ABs), a reversible state associated with persistence. Here, we investigate how early iron deprivation affects transcriptional regulation in *C. trachomatis*. We performed RNA sequencing to compare transcriptional profiles between EBs and RBs and between RBs and ABs induced by iron chelation with 2,2′-bipyridyl at 8 hpi. In EBs, 29% of genes were downregulated and 30% upregulated compared to RBs, revealing extensive transcriptional remodeling. At 24 hpi, ABs displayed downregulation of two-component system (TCS) genes (*atoS*, *atoC*, and *chxR*), while genes encoding inclusion membrane proteins (Incs) and the *trpRBA* operon were upregulated. These findings show how *C. trachomatis* adapts transcriptionally to iron deprivation, revealing stress- and time-dependent changes in metabolism, stress responses, and host–pathogen interactions. Persistence alters the developmental cycle while maintaining inclusion integrity and modulating host interactions, contributing to chronic infection. Uncovering the mechanisms driving persistence may offer crucial insights into chlamydial pathogenesis.

**Supplementary Information:**

The online version contains supplementary material available at 10.1038/s41598-026-48043-y.

## Introduction

*C. trachomatis* is an obligate intracellular bacterium which causes human ocular and sexual infections^1^. Treatment typically involves antibiotics such as azithromycin or doxycycline to relieve symptoms and prevent long-term complications. However, most infections are asymptomatic, contributing to underdiagnosis and an increased risk of complications, such as pelvic inflammatory disease and tubal infertility, and increased transmission to sexual partners^2,3^.

One interesting feature shared by all members of the *Chlamydiota* phylum, including *Waddlia chondrophila,* is their biphasic developmental life cycle: infectious elementary bodies (EB) and replicating reticulate bodies (RB). Extracellular EBs are internalized by host cells via phagocytosis or endocytosis^4,5^. Once internalized, the bacterium resides within a membrane-bound compartment called an inclusion, where it evades host defenses. Inside the inclusion, the EBs differentiate into metabolically active RBs, which divide through binary fission and a budding-like mechanism^6,7^ At the end of the cycle, the RBs differentiate back into EBs, and are released into the extracellular space via exocytosis or extrusion, ready to infect new host cells.

In this study, we compared the transcriptional profiles of EBs and RBs to identify genes preferentially expressed during the infectious and replicative phases of the developmental cycle. Identifying key drivers of bacterial replication may facilitate the discovery of novel drug targets.

Under stressful conditions, *Chlamydiota* members can enter a persistent state characterized by a halt in replication and metabolic activity^8^. This form, known as an aberrant body (AB), is enlarged, non-dividing, and can revert to RBs once stress conditions are lifted^9^. Various stressors such as iron starvation^10^, tryptophan deprivation^6^, IFN-γ^11^, heat-shock^12^, β-lactam antibiotics^13^, phosphomycin^14^, glycopeptides (vancomycin, teicoplanin)^14^, MreB inhibitors^15^ and herpes simplex virus (HSV) co-infection^16^ have been identified as triggers of transition into AB, which are characteristic of persistence *in* *vitro*. While most evidence for AB formation derives from in vitro studies, persistence-like states have been observed in *Chlamydiales* species infecting animals^17,18^, whereas observations from human infections remain limited^19–21^.

Although environmental stimuli triggering persistence are well characterized, the molecular mechanisms driving the transition from the replicative RBs to the persistent ABs remains poorly understood. Transcriptomic analyses revealed that *C. trachomatis* exhibits distinct gene expression profiles depending on the specific stressor. Exposure to penicillin alters expression of genes involved in cell division and peptidoglycan biosynthesis, contributing to AB formation^22^. Heat shock induces chaperone proteins and stress response regulators to protect proteins from damage^23^. Finally, co-infection with HSV has been shown to promote persistence by altering host immune responses and bacterial gene expression^16^.

Nutrient starvation, especially of iron and tryptophan, triggers coordinated transcriptional responses that promote persistence^24–26^. For example, IFN-γ–mediated tryptophan limitation results in upregulation of the *trpRBA* operon to support tryptophan synthesis. The YtgR repressor, which regulates both the *ytgABCD* iron acquisition operon and the *trpRBA* operon, integrates signals from both iron and tryptophan availability. Under iron-limiting conditions, loss of YtgR’s [Fe^2^⁺] cofactor relieves repression of *ytgABCD*, promoting iron uptake. Iron depletion also impairs the activity of iron-dependent enzymes IspH and IspG in the MEP pathway, disrupting peptidoglycan synthesis and division, thereby contributing to persistence^27^. This dual regulation enables continued *trpRBA* expression under tryptophan- or iron-starved conditions while maintaining repression when both nutrients are sufficient. *C. trachomatis* normally obtains iron from their eukaryotic host, often through interactions with host proteins such as transferrin and lactoferrin^25,28^.

Iron chelators such as 2,2′-bipyridyl (BPDL) or deferoxamine sequester free iron ions (Fe^2^⁺ and Fe^3^⁺) in the extracellular environment, thereby reducing iron bioavailability and disrupting bacterial iron homeostasis^29^. This disruption is detrimental for *C. trachomatis*, as iron serves as an essential cofactor for numerous bacterial enzymes involved in vital processes including DNA synthesis, respiration, and cell division^30^. BPDL has demonstrated greater efficacy than deferoxamine in inhibiting *C. trachomatis* development, inducing the AB morphology characteristic of persistence, and eliciting a pronounced iron starvation transcriptional response in both the host and the bacterium^30^.

A genome-wide transcriptional response to iron starvation in *C. trachomatis* was previously characterized by Brinkworth et al*.*, who analyzed short-term (3 or 6 h) BPDL treatments initiated at 12 hpi^31^. Their study revealed that the response to iron limitation is highly dynamic, varying with both the duration of stress exposure and the bacterium’s developmental stage.

Furthermore, a recent work from the same group has shown that host cell responses can amplify nutritional stress and contribute to persistence in *C. trachomatis*^25^. Using a BPDL treatment initiated at 8 hpi, (a condition referred to as BPD16 based on treatment duration), this study demonstrated that iron chelation activates host pathways that impose additional nutritional stresses, including GTP limitation, which reinforces bacterial persistence, amplifying the direct effects of iron deprivation on *C. trachomatis*. These findings indicate that iron starvation-induced persistence arises from the combined effects of direct bacterial iron limitation and host-mediated restriction mechanisms.

In the present study, we conducted a comparative transcriptomic analysis of *C. trachomatis* RBs and ABs following BPDL treatment initiated at 8 hpi and maintained for 16 h. We specifically focused on the bacterial transcriptional response and directly compared RBs and ABs, providing genome-wide insight into bacterial-intrinsic regulatory mechanisms during iron starvation-induced persistence. This earlier induction of persistence, compared to prior studies, had significant implications, particularly regarding the regulation of inclusion membrane proteins (Incs)^21^.

Incs are secreted by *C. trachomatis* via the type III secretion system (T3SS), a needle-like apparatus that enables the bacterium to inject effector proteins into the host cell cytosol during cell entry and once within the inclusion. Within the inclusion membrane, Incs are oriented, so their hydrophobic domains face the host cytoplasm, allowing them to interact with host proteins. These interactions regulate vesicular trafficking, nutrient acquisition, inclusion stability, and host immune modulation. Through these functions, Incs help create a stable intracellular niche that promotes bacterial replication and persistence under stress^32^.

The *incDEFG* operon encodes four Inc proteins (IncD, IncE, IncF, and IncG) with distinct functions^33^. IncD binds the ceramide transporter CERT, redirecting ceramide from the ER to the inclusion membrane, which is important for membrane biogenesis^34^. IncE helps stabilize the inclusion and recruits SNX5/6 trafficking complexes^35^. IncF acts as a scaffolding protein that coordinates interactions among other Incs^36^. IncG modulates host cell signaling to suppress apoptosis through interactions with host protein 14–3-3β^37^. Importantly, IncG expression varies under different stress conditions. Expression is detectable following treatment with ampicillin, sucrose starvation, or BPDL, but is absent when the T3SS is inhibited by C1^38^, when MreB is inhibited by MP265^6^, or following IFN-γ or azithromycin treatment^21^. In addition, persistent *C. trachomatis* can alter the expression and localization of mid- and late-cycle Incs such as IncA and CT813, disrupting normal inclusion-host interactions^21^.

Another important stress response mechanism that *C. trachomatis* uses to adapt and survive within the host cell environment, is the two-component system (TCS). Normally, bacteria use their TCSs to sense and respond to their environment^39^. A typical bacterial TCS consists of two fundamental components: a histidine kinase (HK), which detects environmental signals such as temperature, pH, or nutrient fluctuations, and a response regulator (RR), which functions primarily as a transcription factor. Upon sensing a stimulus, the HK undergoes auto-phosphorylation at a conserved histidine residue, subsequently transferring the phosphoryl group to the RR. This phosphorylation induces a conformational change in the RR, enabling it to bind specific DNA sequences and modulate the expression of target genes^39–41^.

In our study, we aimed to characterize the bacterial transcriptional programs associated with iron starvation-induced persistence in *C. trachomatis* using RNA-seq. By comparing replicative and persistent developmental forms following BPDL treatment initiated at 8 hpi, we sought to define bacterial-intrinsic regulatory pathways linked to persistence. We selected this specific timepoint to align with our previous RNA-seq analysis of persistent *W. chondrophila*, a *Chlamydia*-related bacterium and to allow a direct comparison of persistence mechanisms across related organisms^42^.These analyses provide a framework for understanding how *C. trachomatis* adapts its gene expression to nutrient limitation and may inform future studies of chronic infection and therapeutic intervention.

## Results

### Transcriptomic analysis of the *C. trachomatis* developmental stages using RNA-seq

**Fig. 1:**
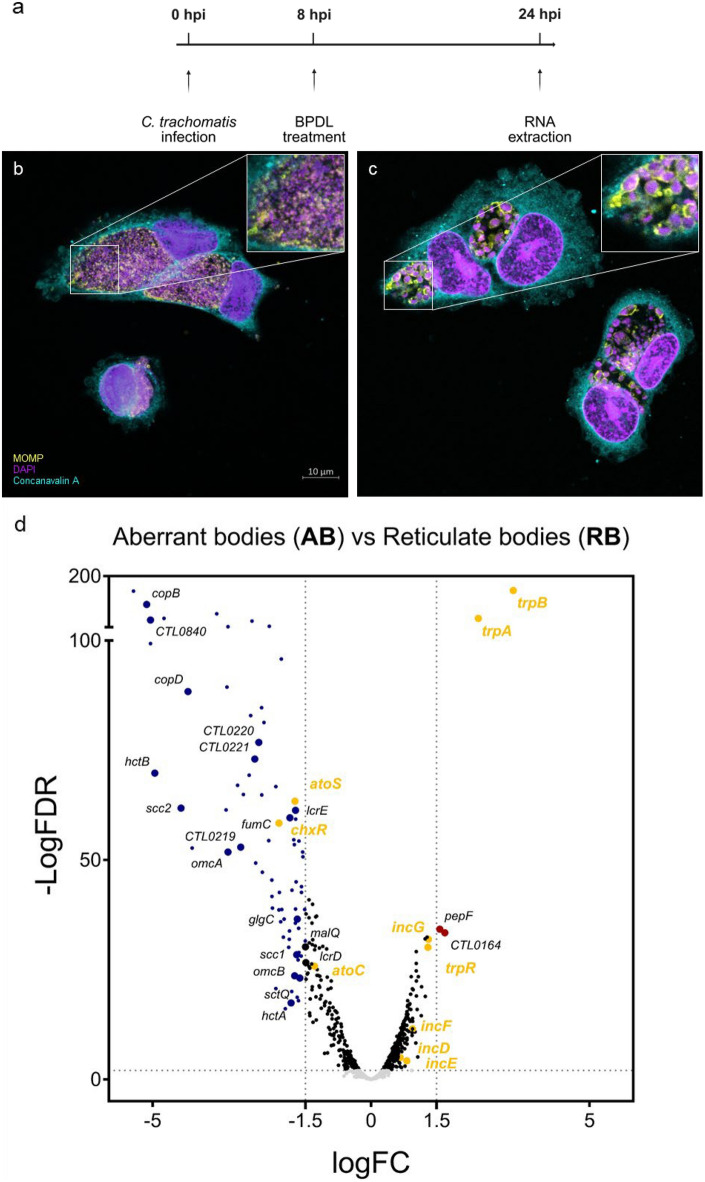
RNA-seq experimental design* C. trachomatis* developmental forms and differential gene expression. (**a**) Schematic of the experimental timeline. BPDL was added to HEp-2 cells infected with *C. trachomatis* 8 hpi, at a concentration of 100 μM and gene expression was measured at 24 hpi. (**b**-**c**) HEp-2 cells were infected with *C. trachomatis* and RNA was extracted from predominately RBs (**b**) or ABs (**c**) at 24 hpi. ABs were induced by BPDL treatment at 8 hpi. At 24 hpi, cells were fixed and labeled with Concanavalin A (turquoise), DAPI (purple), and MOMP (yellow). Scale bar: 10 µm. (**c**) Volcano plot showing differential gene expression between ABs and RBs. Each point represents a gene averaged across three replicates. Genes with FDR ≤ 0.01 and log₂ FC ≤ –1.5 or ≥ 1.5 are shown in blue (downregulated) and red (upregulated), respectively. Genes in yellow belong to operons with one or more, strongly differentially expressed genes: TCS genes, the *trpRBA* operon, and the *incDEFG* operon, which are characterized further on.

A comprehensive transcriptomic analysis of *C. trachomatis* was performed using RNA-seq on cDNA libraries derived from total RNA. To detect and quantify differentially expressed transcripts corresponding to the three developmental stages (EBs, RBs, and ABs), infected cells were lysed at different time points after infection and total RNA was extracted. Immunofluorescence staining validated these time points, confirming that EBs, RBs, or ABs were the predominant form at each sampling time. HEp-2 cells were infected with *C. trachomatis* at an MOI of 1 and collected at 24 hpi for RBs and 72 hpi for EBs (Fig. 1a-b and Supplementary Fig. 1a).

Infected cells were treated with the iron chelator BPDL to induce ABs^10^. BPDL was added at 8 hpi and RNA was collected at 24 hpi (Fig. 1c). BPDL was used at 100 µM, a concentration previously shown to not significantly affect host cell viability^43,44^.

Rarefaction analysis showed that gene detection plateaued across all samples (Supplementary Fig. 2a), indicating sufficient sequencing depth. Between 4.0 and 15.1 million reads per sample, corresponding to 17.44% to 80.95% of total reads, mapped to the *C. trachomatis* genome.

Expression profiles across the three replicates were highly correlated (Pearson r > 0.98; Supplementary Fig. 2b). PCA revealed clear clustering with distinct separation between EB, RB, and AB samples (Supplementary Fig. 2c). Comparing EBs and RBs, 525/888 open reading frames (ORFs) (59.12%) were differentially expressed (29% downregulated, 30% upregulated, FDR ≤ 0.01; p ≤ 0.05). Among them, 9 genes were downregulated with a logFC ≤ –1.5 between the 2 stages, and 21 were upregulated with a logFC ≥ 1.5 (Supplementary Table 1).

Comparison of ABs and RBs showed 558/888 ORFs (62.83%) were differentially expressed (28% downregulated and 34% upregulated). Among them, 80 genes were downregulated with a logFC ≤ –1.5 between the 2 stages, and 4 were upregulated with a logFC ≥ 1.5 (Supplementary Table 2).

Finally, the analysis between EBs and ABs resulted in significant differential expression of 56.19% of the *C. trachomatis* genome, corresponding to 499/888 ORFs (26% downregulated, 30% upregulated, FDR ≤ 0.01; p ≤ 0.05). In this comparison, 5 genes were downregulated with a logFC ≤ –1.5 between the 2 stages, and 51 were upregulated with a logFC ≥ 1.5 (Supplementary Table 3).

### Functional enrichment analysis of ABs vs RBs

To identify patterns in the regulation of different functional pathways, we performed COG-based enrichment analysis across all comparisons (Supplementary Fig. 3a–c). In ABs vs RBs, genes involved in transcription and translation appeared predominantly upregulated, while those in signal transduction were mostly downregulated. Key genes are highlighted in the volcano plot (Fig. 1d). In EBs vs RBs, genes related to amino acid transport and metabolism, as well as translation, ribosomal structure and biogenesis, were upregulated, whereas signal transduction mechanisms were downregulated. In contrast, EBs vs ABs showed upregulation of signal transduction mechanisms and downregulation of genes involved in coenzyme transport and metabolism, along with translation-associated functions. Volcano plots for these comparisons are shown in Supplementary Fig. 1b–c.

### Transcriptional profile of *C. trachomatis* during persistence

In ABs vs RBs, four genes were strongly upregulated in ABs: *trpB* (*CTL0423*, log₂FC = 9.6), *trpA* (*CTL0424*, log₂FC = 5.5), *pepF* (*CTL0367*, log₂FC = 3.0), and *CTL0164* (log₂FC = 3.2). *pepF* encodes an oligopeptidase, while *CTL0164* may be secreted by the Sec system and T2SS, although its function remains unclear^45^. *trpB* and *trpA* belong to the tryptophan operon. The operon repressor *trpR* (CTL0422) was also significantly upregulated (log₂FC = 2.5).

Conversely, several genes were downregulated in ABs when compared to RBs, including metabolic enzymes such as *malQ* (CTL0342, log₂FC = –2.8), *glgC* (CTL0750, log₂FC = –3.2), and *fumC* (CTL0228, log₂FC = –3.3), as well as virulence-associated genes like the T3SS component *sctQ* (CTL0041, log₂FC = –3.1). *hctA* (CTL0112, log₂FC = –3.6), involved in chromatin condensation, was also repressed.

*omcB* (*CTL0702*, log₂FC = –3.4) and *omcA* (*CTL0703*, log₂FC = –9.7) encoding conserved, cysteine-rich outer membrane proteins of the Chlamydial Outer Membrane Complex (COMC), were among the most downregulated in ABs compared to RBs. These proteins are important for structural integrity and host-cell adhesion during the EB stage^46^. The operon including *malQ*, *scc1* (*CTL0343*, log₂FC = –3.2), *lcrE* (*CTL0344*, log₂FC = –3.6), *lcrD* (*CTL0345*, log₂FC = –2.8) was also downregulated, as was *scc2* (*CTL0839*, log₂FC = –8.4), a type III secretion–associated chaperone. This operon also contains *CTL0840* (log₂FC = –33.1) and the hydrophobic T3SS translocator proteins *copB* (*CTL0841*, log₂FC = –35.1) and *copD* (*CTL0842*, log₂FC = –18.2), which are essential for host-cell infection.

Additionally, the operon comprising *CTL0219* (log₂FC = –7.9), *CTL0220* (log₂FC = –5.9), and *CTL0221* (log₂FC = –6.3) was also downregulated in ABs. CTL0219 interacts with the host protein GCIP, while CTL0221 is likely secreted via T3SS; both contain the DUF720 domain. CTL0220, identified as a T3SS substrate in a heterologous system, showed a similar decrease in expression^47^.

Notably, the *atoS–atoC* two-component system (*CTL0727*, log₂FC = –3.3; *CTL0728*, log₂FC = –1.3) and the atypical response regulator *chxR* (*CTL0894*, log₂FC = –2.1) also showed reduced expression in ABs. *atoS* and *atoC*, which are co-transcribed as part of an operon encoding a classical two-component system (TCS), and *chxR*, an atypical response regulator, were selected for further characterization. These findings prompted investigation into their roles in the transcriptional adaptation of *C. trachomatis* during persistence.

### TCS genes are downregulated following iron starvation

**Fig. 2:**
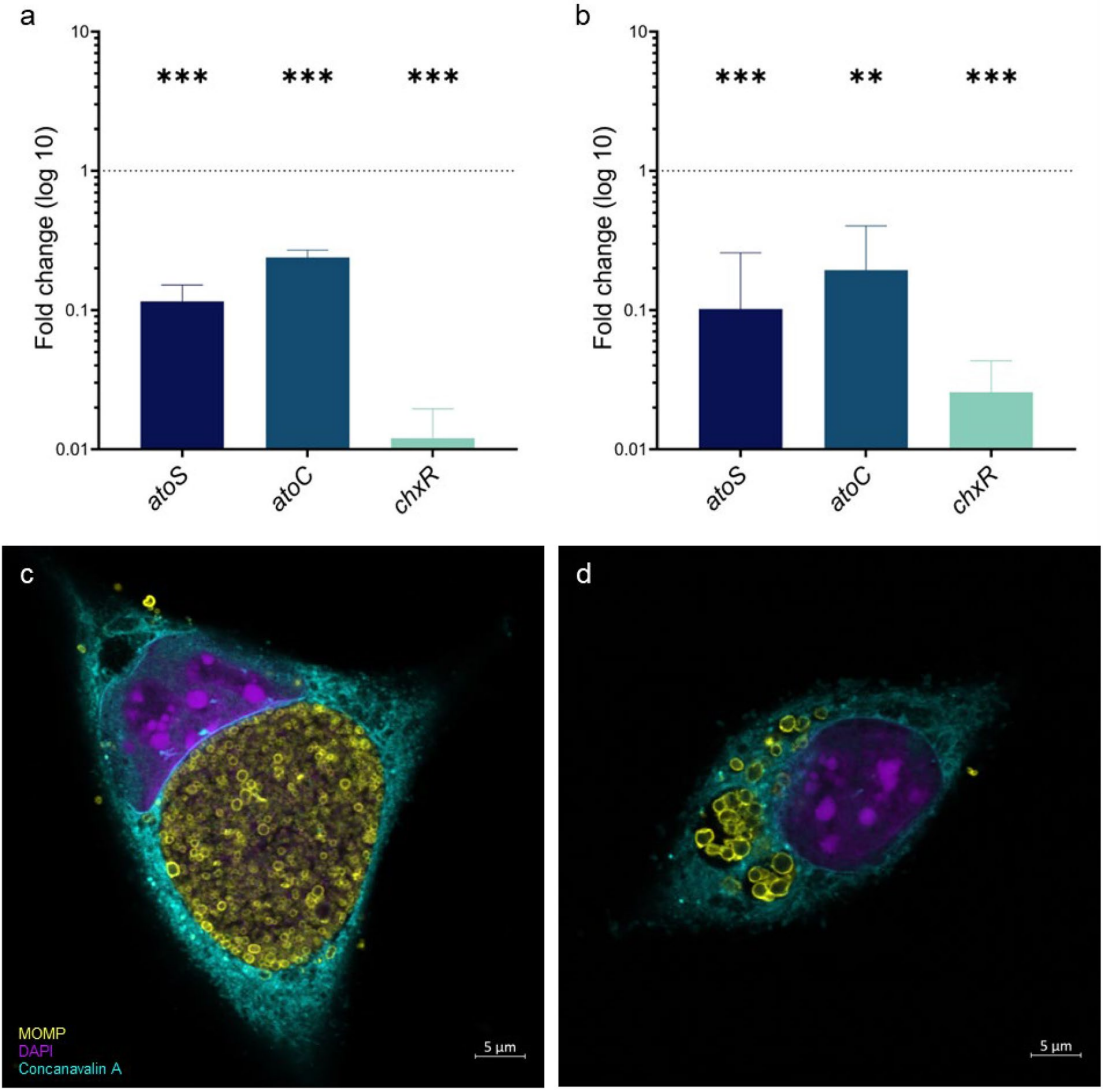
Validation of RNA-seq data and TCS gene downregulation in *C. trachomatis*-infected McCoy cells. Validation of RNA-seq and TCS gene downregulation in *C. trachomatis*-infected cells. (**a**) TCS gene expression at 24 hpi was analyzed by RT-qPCR in HEp-2 cells. Expression levels of *atoS*, *atoC*, and *chxR* were normalized to the expression of the *C. trachomatis* 16S rRNA gene and FC was calculated relative to untreated controls. (**b**) TCS gene expression at 24 hpi was analyzed by RT-qPCR in McCoy cells. Expression levels of *atoS*, *atoC*, and *chxR* were normalized to the expression of the *C. trachomatis* 16S rRNA gene and FC was calculated relative to untreated controls. (**c-d**) Immunofluorescence images showing the morphology of *C. trachomatis* RBs (**c**) and ABs following BPDL treatment (**d**) in McCoy cells. At 24 hpi, cells were fixed and labeled with Concanavalin A (turquoise), DAPI (purple), and MOMP (yellow), prior to observation by confocal microscopy. Scale bar: 5 µm. Statistical significance was assessed using two-sample t test *P < 0.05, **P < 0.01, ***P < 0.001.

The downregulation of TCS genes observed in RNA-seq experiments was first confirmed by RT-qPCR in infected HEp-2 cells treated with BPDL (Fig. 2a). To further assess this differential gene expression, McCoy cells were infected with *C. trachomatis* and treated with BPDL (Fig. 2b) under the same conditions used for RNA-seq (Fig. 1a). Immunofluorescence analysis confirmed the presence of ABs (Fig. 2c-d). RT-qPCR showed that expression of *atoS*, *atoC*, and *chxR* decreased in HEp-2 and McCoy by 10-, 8-, and 50-fold, respectively, at 24 hpi (Fig. 2a-b). These results indicate that the regulatory pattern observed is conserved across both cell lines.

**Fig. 3:**
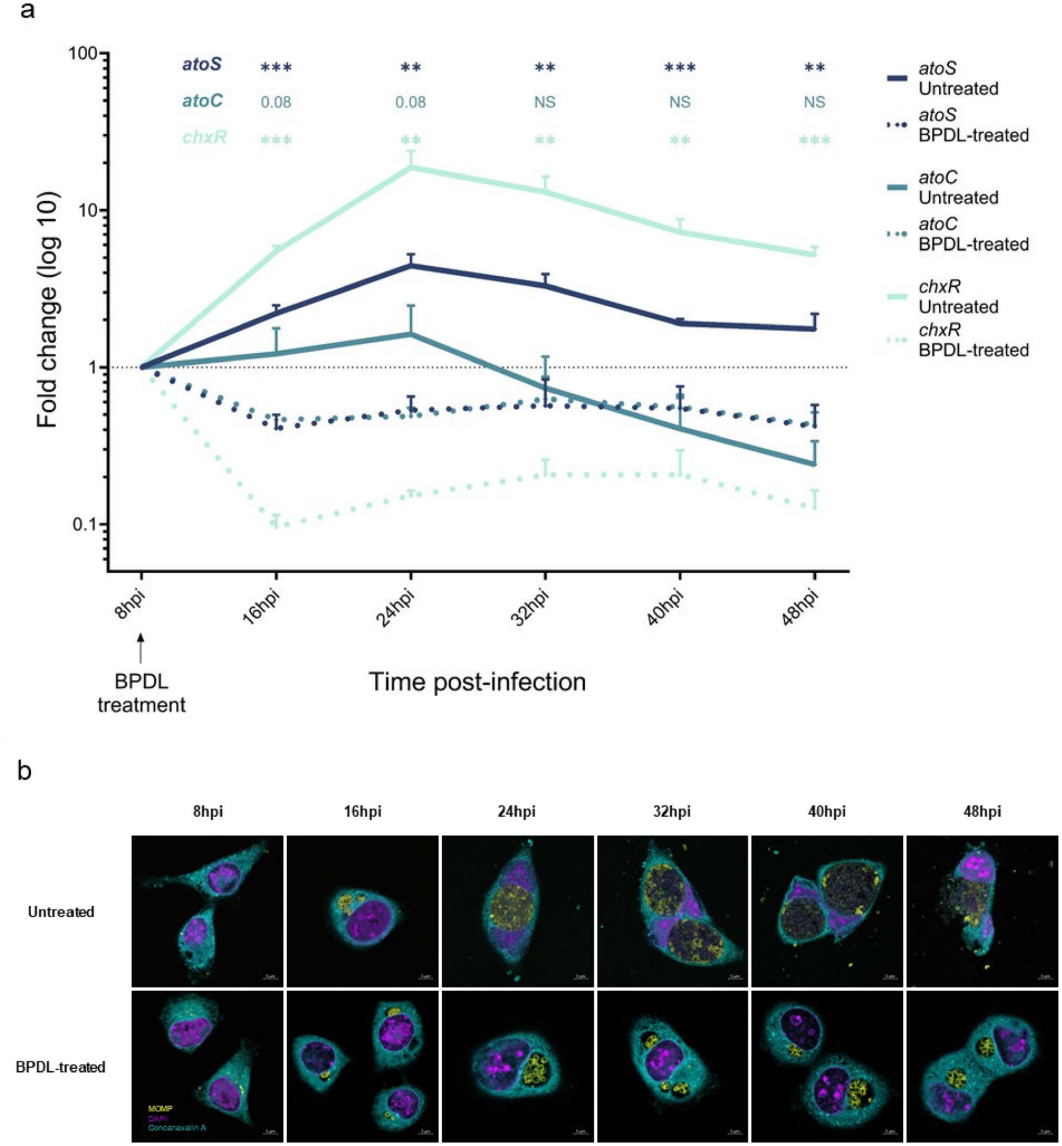
TCS gene expression and morphological changes in *C. trachomatis* under BPDL-induced persistence. (**a**) Time-course analysis of TCS gene expression following *C. trachomatis* infection without (solid lines) or with BPDL treatment (dotted lines). Expression levels of *atoS*, *atoC*, and *chxR* were measured by RT-qPCR, normalized to *16S rRNA*, and calculated relative to untreated samples collected at the corresponding timepoints. Data represent the mean ± SD from three independent replicates and were analyzed by multiple unpaired t-tests. *P < 0.05, **P < 0.01, ***P < 0.001. (**b**) Immunofluorescence images of *C. trachomatis* infected McCoy cells treated with 100 μM BPDL at 8 hpi and fixed at indicated time points. Cells were labeled with Concanavalin A (turquoise), DAPI (purple), and MOMP (yellow), and imaged by confocal microscopy. Scale bar: 5 µm.

We also examined TCS expression across the developmental cycle and following BPDL exposure. Under normal growth conditions, expression of these genes peaked at 24 hpi. After BPDL treatment at 8 hpi, *atoS* and *atoC* decreased 5-fold, and *chxR* 10-fold by 24 hpi (Fig. 3a). Morphologically, bacteria maintained the persistent phenotype, with enlarged forms visible at all time points (Fig. 3c).

### TCS gene expression recovery occurs over 8 h following iron deprivation removal

**Fig. 4:**
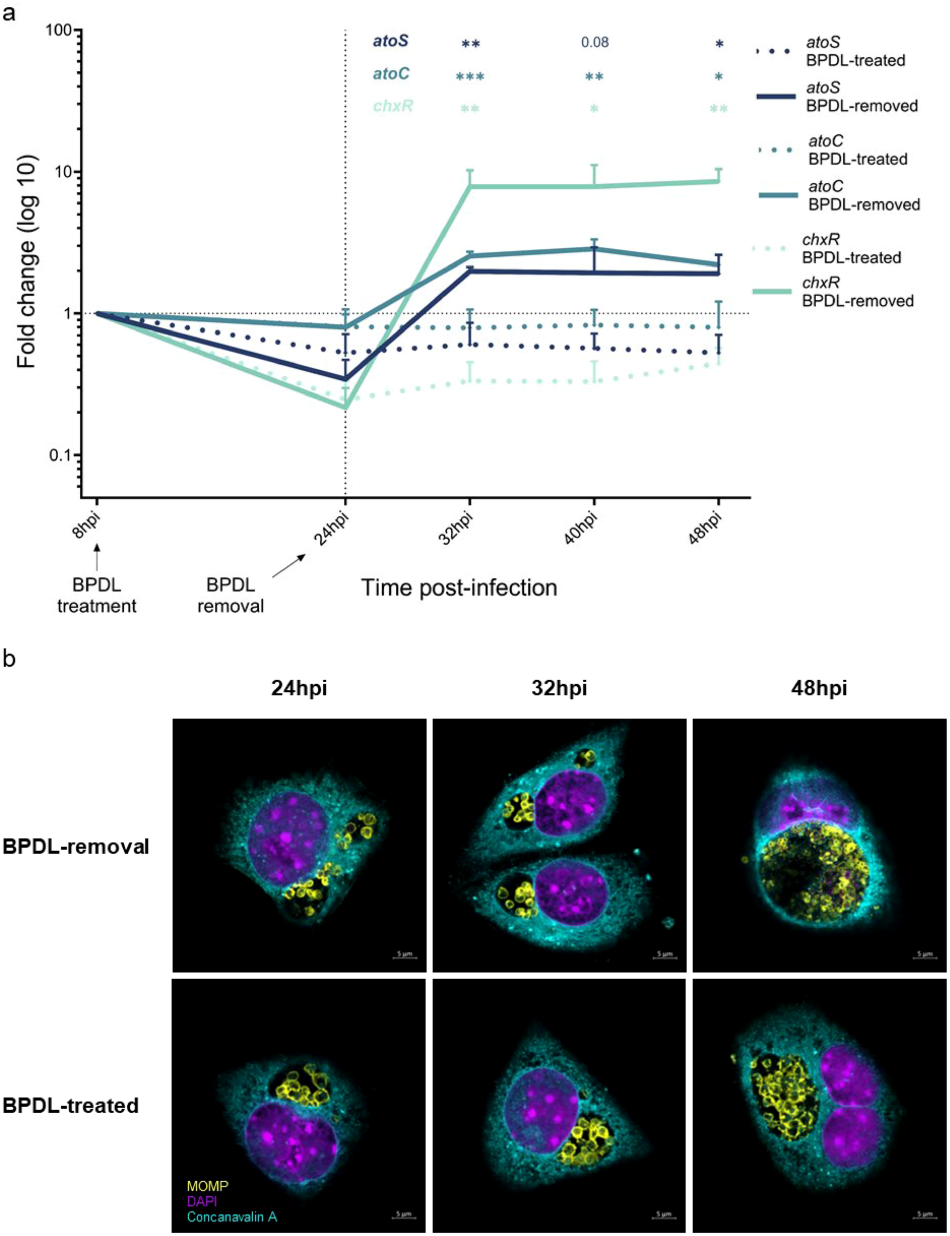
Time-course of TCS gene expression and morphological evolution after BPDL removal. (**a**) Time-course of TCS gene expression in McCoy cells infected with *C. trachomatis* and treated with 100 μM BPDL at 8 hpi. At 24 hpi, BPDL was removed by three washes with fresh DMEM + 10% FBS. Solid lines represent gene expression after BPDL removal; dotted lines represent continued treatment with BPDL. RNA was collected every 8 h from 24 hpi onwards. Expression levels of *atoS*, *atoC*, and *chxR* were measured by RT-qPCR, normalized to *16S rRNA*, and calculated relative to untreated samples collected at the corresponding timepoints. Data represent the mean ± SD from three independent replicates and were analyzed by multiple unpaired t-tests. *P < 0.05, **P < 0.01, ***P < 0.001. (**b**) Immunofluorescence images of infected McCoy cells following BPDL removal at 24 hpi as described in (**a**). Cells were fixed every 8 h and stained with Concanavalin A (turquoise), DAPI (purple), and MOMP (yellow). Scale bar: 5 µM.

Persistence is reversible; when stressors are removed, bacteria can transition from ABs back to RBs and resume replication. To assess TCS gene expression changes after stress removal, media was washed out, three times, at 24 hpi to remove residual BPDL. Bacteria continuously exposed to BPDL showed no changes in TCS gene expression over time. However, following BPDL removal, *atoS*, *atoC*, and *chxR* expression increased, reaching 2-, 2.5-, and 7.8-fold upregulation, respectively, at 32 hpi (8 h post-removal) (Fig. 4a), suggesting stress removal triggers TCS gene expression reactivation.

Immunofluorescence imaging performed alongside genes analysis showed no morphological difference 8 h after BPDL removal (32 hpi) (Fig. 4b). However, at 40 h post-removal, bacteria no longer exposed to BPDL showed a mix of RBs and enlarged ABs, indicating transition back to active growth. In contrast, bacteria still exposed to BPDL remained persistent. These findings indicate TCS gene expression changes preceded morphological recovery and that stress removal enables replication.

### TCS gene downregulation is stressor-specific

**Fig. 5:**
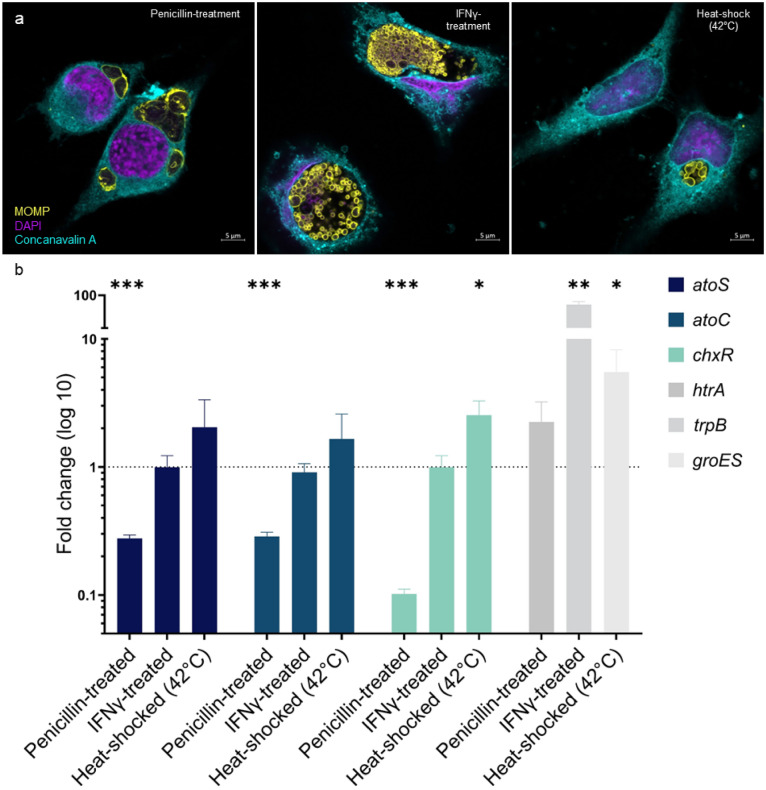
TCS gene expression and morphology of persistent *C. trachomatis* varies with stress type. (**a**) Immunofluorescence images showing *C. trachomatis* morphology under stress-induced persistence. Left: penicillin (5 U/mL) added at 8 hpi; samples fixed at 24 hpi. Middle: IFN-γ (5 µg/mL) added at 8 hpi and again at 12 hpi; samples fixed at 24 hpi. Right: heat shock at 42 °C applied at 8 hpi; samples fixed at 13 hpi (5 h post-treatment). Cells were stained with Concanavalin A (turquoise), DAPI (purple), and MOMP (yellow) and visualized by confocal microscopy. Scale bar: 5 µm. (**b**) McCoy cells were infected with *C. trachomatis* and treated with either penicillin (5 U/mL), IFN-γ (5 µg/mL), or heat shock (42 °C, 5 h). TCS gene expression was analyzed by RT-qPCR at 24 hpi for penicillin- and IFN-γ-treated cells (16 h post-treatment), and at 13 hpi for heat-shocked cells (5 h post-treatment). Expression levels of *atoS*, *atoC*, and *chxR*, together with the controls *htrA* (penicillin), *trpB* (IFN-γ), and *groES* (heat-shock), were normalized to *C. trachomatis* 16S rRNA gene expression and calculated relative to untreated controls. Data represent mean ± SD from three independent replicates. Statistical significance was determined using a two-sample t-test: *P < 0.05, **P < 0.01, ***P < 0.001.

To determine whether TCS gene downregulation was specific to iron starvation or if it is a general response to stress, we examined TCS expression under various stressors that induce AB formation (Fig. 5a). *C. trachomatis*-infected cells were treated with penicillin, IFN-γ, or were heat-shocked (5 h at 42°C) at 8 hpi. Morphological analysis showed variable AB phenotypes, consistent with Scherler et al.^14^, the AB phenotype is known to vary depending on the stressor applied. Penicillin-induced ABs appeared larger than those from BPDL, IFN-γ, or heat shock. Despite these differences, all stressors caused bacterial enlargement and replication arrest, consistent with persistence.

To assess whether TCS gene regulation varies by stress type, we measured *atoS*, *atoC*, and *chxR* expression under each condition. Penicillin strongly downregulated all three genes by 5- to 10-fold relative to untreated controls. IFN-γ had no effect, whereas heat shock caused a 2.5-fold increase in *chxR* only (Fig. 5b). These patterns mirrored the morphological diversity of ABs, with all stressors inducing bacterial enlargement and replication arrest, and penicillin-induced ABs larger than those generated by BPDL, IFN-γ, or heat shock (Fig. 5a). As controls, we also assessed *htrA*, *trpB*, and *groES*, which were previously shown to be upregulated under penicillin^8^, IFN-γ^48^, and heat-shock^49^, respectively.

### Homologous TCS genes in *W. chondrophila* are downregulated during persistence

**Fig. 6:**
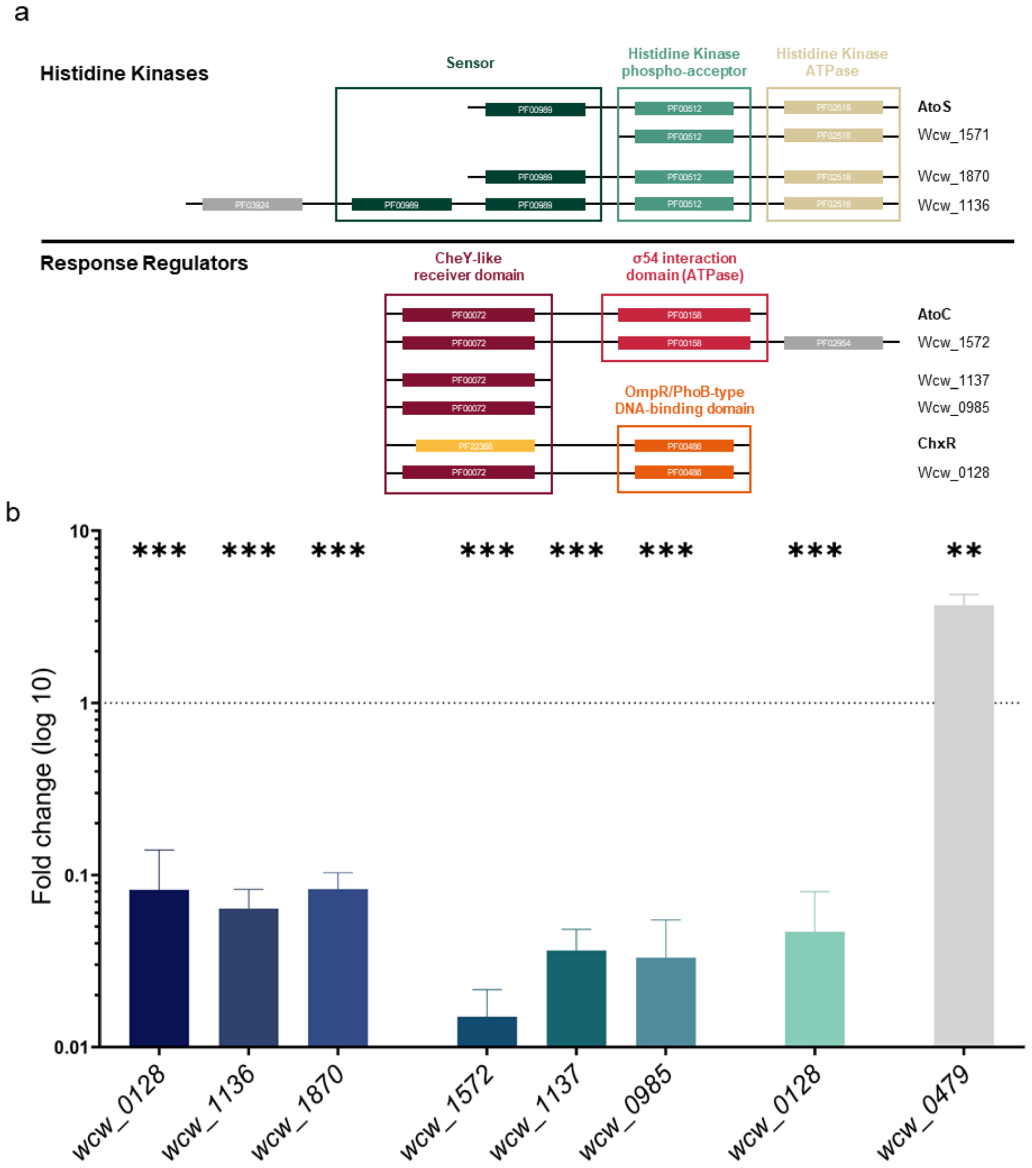
Functional domains of TCS genes and expression of *W. chondrophila* homologs under BPDL treatment. (**A**) Domains are organized by their roles within canonical TCS signaling. Sensor domains (PF00989, PF09394, PF00998) detect environmental or intracellular signals. Histidine kinase phospho-acceptor domains (PF00512) harbor the conserved histidine residue that undergoes autophosphorylation in response to stimuli, a process powered by the histidine kinase ATPase domain (PF02518). CheY-like receiver domains (PF00072, PF12238) are found in response regulators and receive the phosphoryl group from the histidine kinase. The σ^54^ interaction domain (PF00158) enables interaction with σ^54^ RNA polymerase to initiate transcription, while the OmpR/PhoB-type DNA-binding domain (PF00486) mediates the transcriptional regulation of target genes. (**B**) Expression levels of TCS genes in *W. chondrophila* following BPDL treatment. *W. chondrophila* infected cells were treated with BPDL at 8 hpi, and samples were harvested at 24 hpi. Gene expression was measured using RT-qPCR. FC is calculated relative to the untreated control and samples were normalized based on the *W. chondrophila* 16S rRNA gene expression. As a control, the gene *wcw_0479* was also measured. Data are presented as the mean ± SD from three independent replicates. Statistical significance was assessed using two-sample t-tests. *P < 0.05, **P < 0.01, and ***P < 0.001.

To assess whether this regulatory pattern is conserved among Chlamydiae, we examined homologous TCS genes in *W. chondrophila*, a related species that also undergoes persistence under iron starvation. Consistent with recent findings that highlight the diversification and functional relevance of *atoS*, *atoC*, and *chxR* across Chlamydiae^50^, sequence homology analysis revealed seven predicted homologs of these regulators in the *W. chondrophila* genome^51^. Functional domain architecture was assessed using ChlamDB^51^, and protein relationships were visualized (Fig. 6a). Among predicted histidine kinases, *wcw_1571* was the closest *atoS*, homolog, though it lacks a sensor domain. It is located next to *wcw_1572*, a predicted response regulator with features similar to *atoC*. This regulator does not possess a canonical DNA-binding domain but instead contains a domain required for σ^54^-dependent transcriptional activation^52,53^. Other kinases, such as Wcw_1870 and Wcw_1136, contain intact sensor domains. Only two gene pairs, *wcw_1571/wcw_1572* and *wcw_1136/wcw_1137*, appear to be organized in operons. A *chxR* homolog was also identified based on sequence and domain similarity. Unlike in *C. trachomatis*, where ChxR lacks the conserved aspartate residue typical of response regulators, the *W. chondrophila* homolog retains it.

To determine whether these genes respond to iron starvation, expression of the predicted homologs was measured by RT-qPCR in *W. chondrophila*–infected McCoy cells treated with BPDL at 8 hpi and harvested at 24 hpi. All seven were downregulated between 10- and 50-fold compared to untreated controls (Fig. 6b). We included *wcw_0479* as a control because it has been reported to be upregulated under BPDL treatment in previous studies^42^. Although these TCS homologs exhibit similar downregulation during persistence in both *C. trachomatis* and *W. chondrophila*, a previously published study comparing global gene expression in *W. chondrophila*^42^ RBs and ABs revealed a transcriptional landscape that differs markedly from *C. trachomatis* (Fig. 1d), emphasizing species-specific regulatory responses.

### *trpRBA* and *incDEFG* operons are upregulated during BPDL-induced persistence

In *C. trachomatis*, most differentially expressed genes, identified by RNA-seq of ABs compared to RBs, were downregulated, but some were significantly upregulated (Fig. 1d). Among the latter, *trpB* and *trpA*, involved in tryptophan metabolism, were the most upregulated (9.6- and 5.5-fold, respectively). *trpR* was also among the top upregulated genes, with a 2.5-fold increase.

Two genes, *incG* and *incF*, part of the *incDEFG* operon, were also significantly upregulated (2.5- and 1.9-fold, respectively). *incG*, which encodes an inclusion membrane protein involved in membrane stability and host–pathogen interactions^46^, was also one of the most upregulated genes.

**Fig. 7:**
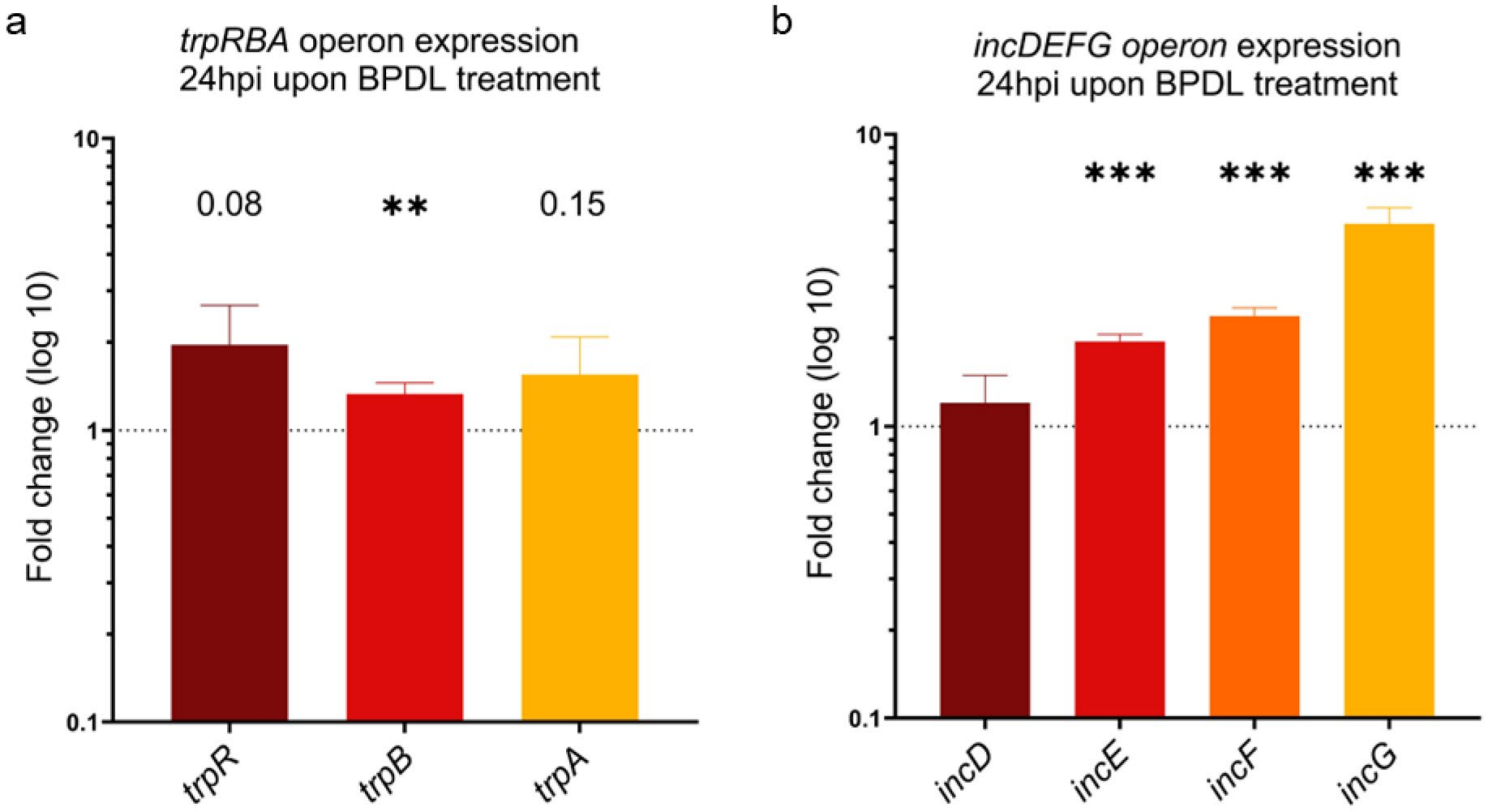
Comparative analysis of *trpRBA* and *incDEFG* gene expression during BPDL-induced persistence. McCoy cells were infected with *C. trachomatis* and ABs were induced with the addition of BPDL at 8 hpi. Expression of *trpRBA* (**a**) and *incDEFG* (**b**) was then assessed at 24 hpi by RT-qPCR. Gene expression levels were normalized to the *C. trachomatis* 16S rRNA gene expression and calculated relative to untreated controls at 24 hpi. Data represent mean ± SD from three independent replicates. Statistical significance was determined by two-samples t-test: *P < 0.05, **P < 0.01, ***P < 0.001.

To validate these transcriptomic results, RT-qPCR was performed on *C. trachomatis*–infected cells treated with BPDL and compared to untreated controls. *trpR*, *trpB*, and *trpA* showed increased expression of approximately 2-, 1.3-, and 1.5-fold, respectively, at 24 hpi in ABs (Fig. 7a). Similarly, *incDEFG* were upregulated under BPDL treatment, with *incG* increasing 5-fold and *incD*, *incE*, and *incF* showing 1.2-, 1.9-, and 2.3-fold increases, respectively (Fig. 7b).

## Discussion

The ability of *C. trachomatis* to transition into a persistent state under stress conditions and the interactions between the pathogen and its environment are largely not understood. Therefore, we aimed to investigate the molecular mechanisms underlying the regulation of persistence, particularly by focusing on the transcriptional changes in response to iron starvation induced by the iron chelator BPDL.

Our unbiased transcriptomic analysis of *C. trachomatis* RBs and ABs by RNA-seq allowed a direct comparison of gene expression profiles. We observed significant downregulation of *hctB*, further supporting previous findings that *hctB*/Hc-2 repression is a reliable marker of persistence^54^. Additionally, under AB-inducing conditions, *atoS*, *atoC*, and the atypical response regulator *chxR*, key components of the *C. trachomatis* TCS, were expressed at reduced levels (Fig. 2c).

TCS genes typically coordinate bacterial responses to environmental cues such as nutrient availability and stress^30^. These systems function through a sensor kinase (such as *atoS*) detecting external signals and a response regulator (such as *atoC*) altering gene expression to mediate adaptive responses. However, bioinformatic analysis did not identify any predicted transmembrane regions in AtoS or *W. chondrophila*-related HKs. In the absence of predicted membrane domains, we speculate that this system may monitor intracellular conditions rather than extracellular ones, suggesting that AtoS responds to changes within the bacterial cytosol rather than at the bacterial surface. In *E. coli*, there are 29 TCSs^55^ including one composed of AtoS histidine kinase (HK) and AtoC RR^56^. Expression of these proteins regulates flagellar motility by the modulation of genes involved in flagellum assembly and function. This motility enables *E. coli* to respond to environmental changes by facilitating movement toward favorable conditions and away from harmful stimuli^57,58^. In contrast, *Chlamydia* species lack flagella as they are adapted to an intracellular niche, where motility is unnecessary for survival. Despite the loss of a flagellum, *Chlamydia* species have retained the homologous AtoS/AtoC as their unique, complete TCS^59,60^.

Importantly, the *atoS/atoC* TCS, also known as *ntrB/C* or *ctcB/C*, is predicted to regulate σ^54^-dependent transcription in *C. trachomatis*. AtoC likely acts as a bacterial enhancer-binding protein (bEBP) activating σ^54^ (RpoN), driving expression of membrane remodeling and T3SS genes during RB-to-EB conversion. This places *atoS/atoC* upstream of σ^54^ activity, linking environmental sensing to late developmental events and infectious particle formation^52,53,61^.

In canonical TCSs, RRs are activated by phosphorylation via HKs at a conserved aspartate residue. However, *C. trachomatis* ChxR is an atypical RR, that lacks this conserved residue and is structurally poised to bind DNA without requiring phosphorylation^60,62^. Previous studies demonstrated that RR downregulation reduces signal transmission, potentially acting as an ‘off’ switch^41,63,64^.

Moreover, ChxR regulates transcription of virulence genes essential for in vivo infection^65^. In particular *CTL0260* (*CT005*), *CTL0828* (*CT565*), and *CTL0064* (*CT695*), which are expressed early^48,65^, suggesting ChxR influences initial host colonization despite its late expression. *CTL0260* (*CT005*) and *CTL0466* (*CT214*) encode inclusion membrane proteins, while *CTL0063* (*CT694*) and *CTL0064* (*CT695*) are T3SS components, highlighting the role of ChxR in host–pathogen interaction. These observations support a model where ChxR acts late in the developmental cycle to regulate virulence factors essential for early infection^65^.

Downregulation of the *C. trachomatis* TCS genes during iron starvation suggests a shift to a metabolically dormant state where downregulation of replication and metabolism genes expression decreases to preserve energy and resources. When the stress is removed, transcription of TCS genes resumes (Fig. 4a). This reversibility is a key feature of bacterial persistence, highlighting the dynamic nature of the bacterial response.

Our data suggests that TCS gene downregulation is not a universal persistence marker but specific for certain stressors such as iron starvation and β-lactam antibiotics, whereas immune and heat stress induce different transcriptional responses (Fig. 5b). Additionally, a previously published transcriptomic analysis also found that TCS genes were not differentially expressed upon IFN-γ treatment of *C. trachomatis* infected cells compared to untreated^48^. Noteworthy, the downregulation of TCS genes observed under both iron starvation and β-lactam stress may be linked to perturbation of peptidoglycan biosynthesis or cell division, suggesting that transcriptional regulation of AtoS and AtoC might be altered when cell division processes are impaired. This suggests that *C. trachomatis* employs distinct regulations, dependent on the type of stress. Interestingly, another transcriptomic analysis of heat stress showed *atoS* and *atoC* upregulated after stronger, longer heat exposure than examined here^23^. In their study, the infected cells were exposed to 8 h of heat stress at 45°C, and in turn, *atoS* and *atoC* were upregulated by 2.5- and 2-fold, respectively. However, 45°C does not represent a physiological temperature that host cells would typically be exposed to. Finally, another transcriptomic study of penicillin-induced persistence independently confirmed the downregulation of *atoC*, reporting a 4.5-fold decrease in expression^22^.

Together with other studies^22,23,48^, our results indicate that transcriptional regulation of TCS genes varies with stress type. Penicillin, a β-lactam antibiotic, targets bacterial cell wall synthesis^66,67^ and induces a transcriptional response similar to BPDL treatment, including downregulation of TCS genes. In contrast, IFN-γ and heat-shock activate immune response and protein misfolding pathways, respectively^48,67^, but do not show similar repression of TCS genes. These findings suggest that iron deprivation and penicillin trigger a stress-specific transcriptional program marked by reduced metabolic activity and shift to a quiescent state.

In addition, recent work has demonstrated that BPDL-induced iron starvation not only directly restricts bacterial iron availability but also activates host cell pathways that create additional nutritional stresses, such as GTP limitation, which amplify the direct effects of iron deprivation on *C. trachomatis*^25^. These data support the idea that persistence emerges from coordinated host-mediated and bacterial-intrinsic responses to iron limitation.

To evaluate whether transcriptional responses to BPDL-induced persistence are conserved across different experimental conditions, we compared our results with previously published transcriptomic data. Previously published studies by Brinkworth et al. and Pokorzynski et al. examined gene expression in *C. trachomatis*-infected HeLa cells during persistence induced by BPDL added at 0, 8 or 12 hpi^25,31^. In our study, BPDL was added at 8hpi on infected HEp-2 or McCoy cells. Comparison across datasets further supports the context-dependent nature of chlamydial persistence. Specifically, the *trpBA* operon was consistently upregulated following BPDL treatment in all studies, suggesting that tryptophan biosynthesis is a conserved response to iron starvation. However, differences in the expression of other genes highlight how experimental timing may influence transcriptional outcomes. *nrdA*, a gene involved in ribonucleotide reduction, was significantly upregulated in the 12 hpi BPDL-treated condition from Brinkworth et al. but not in our 8 hpi experiment. Conversely, *glgC*, which encodes a key enzyme in glycogen biosynthesis, was downregulated in our dataset and upregulated in theirs. These discrepancies likely reflect the asynchronous developmental cycle of *C. trachomatis*, where the stage of inclusion maturation at the time of stress exposure influences the pathogen’s transcriptional response^21^ . Between 8 and 12 hpi, inclusion biogenesis is ongoing, and the bacterium actively suppresses host immune detection by reducing the release of immunostimulatory peptidoglycan (PG) muropeptides^52^. The more advanced developmental state at 12 hpi may therefore underlie the transcriptional divergence observed between the two datasets^31^.

To further explore how *C. trachomatis* adapts its transcriptional program during persistence, we compared transcriptional changes under BPDL treatment with those observed in *W. chondrophila* Both species exhibit similar phenotypic responses to iron deprivation, and when considering all differentially expressed genes, no major differences in the number of regulated genes were observed. However, the proportion of differentially expressed genes is lower in *W. chondrophila*, consistent with its genome being approximately twice the size of the *C. trachomatis* genome. When applying the respective cutoffs for significantly differentially expressed genes in response to iron starvation (logFC ± 1.5 for *C. trachomatis*; ± 2 for *W. chondrophila*), more genes appear upregulated in *W. chondrophila* while more genes are downregulated in *C. trachomatis*^42^.

Despite these overall differences, a few homologous genes showed similar expression patterns under iron starvation in both species. These included the stress-related *groEL* (*CTL0365/wcw_1343*) and *groES* (*CTL0366/wcw_1848*) genes and the histone-like protein *hctA* (*CTL0112/wcw_0203*). Beyond these, the TCS genes were the only other group which displayed conserved transcriptional changes. Specifically, the HK gene *atoS* (*CTL0727/wcw_1571*) and the RR gene *chxR* (*CTL0894/wcw_0128*) were downregulated during persistence in both *C. trachomatis* and *W. chondrophila*^42^.

Alongside the upregulation of the *trpRBA* operon, we also observed increased expression of *incG*, which was the fourth most upregulated gene in ABs compared to RBs (Fig. 1d). Since Incs are critical for inclusion formation and maintenance, as well as modulation of host–pathogen interactions, their upregulation during BPDL-induced persistence underscores their importance in this stressed state. Interestingly, the gradient in upregulation across the *incDEFG* operon, following the operon arrangement, may reflect promoter proximity or differences in transcript stability, suggesting that operon architecture could modulate individual gene expression. Consistent with our results, previous transcriptomic analyses reported upregulation of *incG* and *incF* under bipyridyl-induced iron starvation^68^. Noteworthy, this upregulation was not observed with tryptophan-starved bacteria, suggesting that regulation of these Incs may depend on the specific stress encountered. Notably, the TCS genes *atoS* and *atoC* were downregulated under both bipyridyl treatment and tryptophan limitation^68^ supporting the idea that repression of these regulators represents a broader persistence-associated response. Apart from iron depletion, another host defense mechanism against invading pathogens is the limitation of tryptophan. In the study by Riffaud-Widner et al., tryptophan depletion following infection of cells with *C. trachomatis* was also shown to induce changes in inclusion membrane composition by the upregulation of Incs^69^. Additionally, during IFN-γ induced persistence, *C. trachomatis* is unable to acquire new tryptophan due to the expression of indoleamine 2,3-dioxygenase (IDO), an enzyme that catabolizes tryptophan^8,70^. In response, *C. trachomatis* modifies the inclusion membrane with Incs. Notably, increased levels of IncD and IncG have been observed during IFN-γ-induced persistence^69^. These findings demonstrate that host-*C. trachomatis* interactions are maintained, despite an arrest in replication, and that the T3SS that secretes Inc proteins, remains functional during persistence.

It has been proposed that transcriptional and structural changes induced during persistence contribute to immune evasion by reducing the immunogenic profile of *C. trachomatis*. At the same time, the pathogen must counteract host stress responses triggered by environmental insults such as nutrient deprivation or heat shock, both of which can elevate reactive oxygen species (ROS) and induce apoptosis^71,72^. Since premature host cell death compromises bacterial survival, preserving host cell viability is essential for maintaining the intracellular niche. Together, the coordinated regulation of TCS genes, the tryptophan salvage pathway via the *trpRBA* operon, and modulation of inclusion membrane proteins reflects a multifaceted bacterial strategy to survive nutrient deprivation and host immune pressures during persistence. Under BPDL-induced persistence, these factors likely help the bacteria adapt to stress by maintaining inclusion membrane integrity and modulating host immune responses, thereby preserving cellular homeostasis and ensuring continued bacterial development within the host.

While our study provides valuable insights, there are some limitations. For instance, the use of immortalized cell lines such as HEp-2 and McCoy may not fully capture the complex dynamics of natural, primary cell environments. These cell lines can exhibit alterations in key biological processes, including apoptosis and stress response pathways, which may influence how *C. trachomatis* regulates gene expression. Consequently, these cellular differences could impact the expression of the *incDEFG* operon and other persistence-associated genes. Taken together, these limitations highlight the need for further research using primary cells or in vivo models to gain a more accurate understanding of the molecular mechanisms at play. Another important consideration is the timing of sample collection. To compare untreated, persistent forms (ABs) with treated, non-persistent stages (RBs), we selected 24 hpi for BPDL-treated samples. Despite the potential for this time point to include a mix of RBs and emerging EBs, it offered a biologically relevant window to capture the replicative phase prior to late-stage differentiation. This transitional phase may influence gene expression profiles and complicate interpretation. Nevertheless, this time point also balanced the need for a sufficient infection rate and adequate RNA yield, which were critical for downstream transcriptomic analyses.

Future studies should aim to elucidate the precise roles of TCS and Incs in the establishment and maintenance of persistence. Additionally, exploring host-specific factors that influence these pathways will be crucial. Investigating the effects of various stressors on gene expression and persistence will broaden our understanding of the environmental cues that induce the persistent phenotype in *C. trachomatis*. Ultimately, advancing knowledge of these persistence mechanisms may contribute to the development of more effective treatments for chronic chlamydial infections, which remain a significant global health burden.

## Material and methods

### Cell culture, infection and treatment

HEp-2 (ATCC CCL-23), McCoy (ATCC CRL-1696), and HeLa (ATCC CCL-2) cells were maintained in DMEM supplemented with 10% FBS at 37°C with 5% CO₂. For infection, confluent cultures were infected with *C. trachomatis* L2 (ATCC VR-902B) (frozen stock, MOI 1) or a co-culture of *W. chondrophila* (ATCC VR-1471) in amoebae (MOI 1), diluted 1:1000 or 1:100, respectively. *C. trachomatis*-infected cells were centrifuged at 900 × *g* for 15 min; *W. chondrophila*-infected cells were centrifuged as previously described^14,42^. Cells were incubated at 37°C with 5% CO₂ for 30 min (*C. trachomatis*) or 15 min (*W. chondrophila*) to facilitate uptake. Medium was replaced to remove extracellular bacteria. IFN-γ.

Iron starvation was induced at 8 hpi by addition of 100 µM BPDL (*C. trachomatis*) or 75 µM BPDL (*W. chondrophila*) solubilized in ethanol. When required, BPDL was removed at 24 hpi by three washes with DMEM + 10% FBS. Penicillin (5 U/ml; Sigma-Aldrich) was added at 8 hpi and samples collected at 24 hpi. For IFN-γ treatment, HeLa cells were used because human IFN-γ was applied and this cell line provides an appropriate human epithelial model for IFN-γ–induced persistence in *C. trachomatis*. For heat-shock, infected cells were incubated at 42°C at 8 hpi and collected 5 h later.

### Immunofluorescence staining and imaging

Infected cells were fixed in ice-cold methanol for 5 min at room temperature, washed in PBS, and blocked/permeabilized in PBS with 1% BSA, 0.1% saponin, and 0.04% NaN₃ for ≥ 2 h at 4 °C. Coverslips were incubated with goat anti-MOMP (1:500, LifeSpan BioScience) for 2 h at RT, washed, and stained with Alexa Fluor 488–conjugated chicken anti-goat IgG (1:1000, Thermo) containing DAPI (150 ng/ml, Invitrogen). Texas Red–conjugated Concanavalin A (100 ng/ml, Invitrogen) was added to stain host cells. Coverslips were washed and mounted in Mowiol 4–88 (Sigma-Aldrich). Imaging was performed using a Zeiss LSM900 confocal microscope with a 100 × oil objective. Image analysis was done with Zen 3.2.

### RNA extraction

For RNA-seq, HEp-2 cells from a single T25 flask were scraped into culture medium, centrifuged at 1,790 × *g* for 10 min, and the supernatant centrifuged again at 5,000 × *g* for 10 min. Pellets were combined and resuspended in 2.5 ml TRIzol™ (Invitrogen), vortexed, and stored at –20 °C. Samples were thawed, vortexed, and incubated 5 min at RT. Chloroform (500 µl) was added, vortexed (15 s), and incubated 2 min at RT. After centrifugation at 12,000 × *g* for 15 min at 4 °C, the aqueous phase was mixed with 625 µl isopropanol, incubated 10 min at RT, and centrifuged again. RNA pellets were washed twice with 75% ethanol, air-dried, and resuspended in 30 µl DEPC-treated water. DNase digestion was performed with DNase I (Invitrogen) for 30 min at 37 °C, and inactivation reagent was used to terminate the reaction. RNA was stored at –80 °C.

For RT-qPCR, McCoy cells were infected and harvested as above, then processed with RNAprotect (QIAGEN). RNA extraction was performed using a QIAcube and the RNeasy Mini Kit (QIAGEN). cDNA was synthesized using the QuantiTect Reverse Transcription Kit (QIAGEN).

### RNA sequencing

RNA quality was verified using a Fragment Analyzer (Agilent). rRNA was removed using the Ribo-Zero rRNA Removal Kit (Illumina). RNA was quantified with the Qubit™ RNA Broad-Range Assay Kit (Thermo Fisher) and processed by Fasteris SA (Geneva, Switzerland), which performed poly-A negative selection, library prep (Illumina TruSeq Stranded mRNA Library Prep Kit), and sequencing on an Illumina NovaSeq 6000.

### Sequence read processing and gene expression analysis

Reads were trimmed with *trimmomatic*^73^ version 0.36 and mapped to the *C. trachomatis* L2/434/Bu genome (GCA_000068585.1) using *bwa-mem* v0.7.17^74^ Gene counts were obtained using *htseq-count* v0.11.2 (stranded = no). Rarefaction analysis was performed by down-sampling reads from 500,000 to full depth in 500,000-read steps to assess gene detection saturation.

Differential expression was performed using *edgeR* v3.26.5^75^. Library sizes were normalized using TMM via *calcNormFactors*^76^, and low-abundance genes were filtered using *filterByExpr*. Dispersion was estimated with *estimateCommonDisp* and *estimateTagwiseDisp*. Differentially expressed genes were identified using *exactTest*, and significance was defined as FDR < 0.01. Normalized expression was reported as RPKM. Heatmaps were generated using *pheatmap* (log₂ RPKM; complete linkage clustering method^77^. Functional annotations were retrieved from KEGG^78^, ChlamDB^51^.

### RT-qPCR

RT-qPCR was performed using 4 µl of 5 × diluted cDNA, 10 µl iTaq Universal SYBR Green (BioRad), and gene-specific primers (see Appendix). Cycling conditions: 95°C for 3 min; 45 cycles of 95°C for 15 s and 60°C for 1 min; followed by melt curve analysis. Amplification was performed on a QuantStudio Real-Time PCR System (Thermo). Fold changes were calculated using the 2^–∆∆Ct method with *16S rRNA* as endogenous control. Data analysis was performed with Prism v8.0.1 (GraphPad).

## Supplementary Information

Supplementary Information 1

Supplementary Information 2

## Data Availability

The RNA-seq datasets generated and analyzed during the current study are available in the Gene Expression Omnibus (GEO) repository under accession number GSE317337 (https://www.ncbi.nlm.nih.gov/geo/query/acc.cgi?acc=GSE317337).

## References

[1] Li, C., Ong, J., Tang, W. & Wang, C. Editorial: *Chlamydia trachomatis* infection: epidemiology, prevention, clinical, and basic science research. *Front. Public Health* **11**, 1167690 (2023). 36960363 10.3389/fpubh.2023.1167690 PMC10028239

[2] Rodrigues, R., Marques, L., Vieira-Baptista, P., Sousa, C. & Vale, N. Therapeutic options for *Chlamydia trachomatis* infection: present and future. *Antibiotics (Basel)* **11**, 1634 (2022). 36421278 10.3390/antibiotics11111634 PMC9686482

[3] Lane, A. B. & Decker, C. F. *Chlamydia trachomatis* infections. *Dis. Mon.* **62**, 269–273 (2016). 27091634 10.1016/j.disamonth.2016.03.010

[4] Hybiske, K. & Stephens, R. S. Mechanisms of *Chlamydia trachomatis* entry into nonphagocytic cells. *Infect. Immun.* **75**, 3925–3934 (2007). 17502389 10.1128/IAI.00106-07 PMC1952008

[5] Greub, G. & Raoult, D. Crescent bodies of Parachlamydia acanthamoeba and its life cycle within Acanthamoeba polyphaga: an electron micrograph study. *Appl. Environ. Microbiol.* **68**, 3076–3084 (2002). 12039769 10.1128/AEM.68.6.3076-3084.2002 PMC123927

[6] Ouellette, S. P., Karimova, G., Subtil, A. & Ladant, D. *Chlamydia* co-opts the rod shape-determining proteins MreB and Pbp2 for cell division. *Mol. Microbiol.* **85**, 164–178 (2012). 22624979 10.1111/j.1365-2958.2012.08100.x

[7] Ouellette, S. P. et al. Localized cardiolipin synthesis is required for the assembly of MreB during the polarized cell division of Chlamydia trachomatis. *PLoS Pathog.* **18**, e1010836 (2022). 36095021 10.1371/journal.ppat.1010836 PMC9499288

[8] Panzetta, M. E., Valdivia, R. H. & Saka, H. A. Chlamydia Persistence: a survival strategy to evade antimicrobial effects in-vitro and in-vivo. *Front Microbiol.* **12**(9), 3101 (2018). 10.3389/fmicb.2018.03101 PMC6299033 30619180

[9] Wyrick, P. B. Chlamydia trachomatis persistence in vitro: an overview. *J. Infect. Dis.* **201**, S88–S95 (2010). 20470046 10.1086/652394 PMC2878585

[10] Raulston, J. E. Response of *Chlamydia trachomatis* serovar E to iron restriction in vitro and evidence for iron-regulated chlamydial proteins. *Infect. Immun.* **65**, 4539–4547 (1997). 9353031 10.1128/iai.65.11.4539-4547.1997 PMC175652

[11] Regulation of the mitochondrion-fatty acid axis for the metabolic reprogramming of chlamydia trachomatis during treatment with β-lactam antimicrobials. mBio https://journals.asm.org/doi/10.1128/mbio.00023-21 10.1128/mBio.00023-21 PMC8092193 33785629

[12] Kahane, S. & Friedman, M. G. Reversibility of heat shock in *Chlamydia trachomatis*. *FEMS Microbiol. Lett.* **97**, 25–30 (1992). 10.1016/0378-1097(92)90358-u 1330821

[13] Kintner, J., Lajoie, D., Hall, J., Whittimore, J. & Schoborg, R. V. Commonly prescribed β-lactam antibiotics induce *C. trachomatis* persistence/stress in culture at physiologically relevant concentrations. *Front. Cell. Infect. Microbiol.* **4**, 44 (2014). 24783061 10.3389/fcimb.2014.00044 PMC3990100

[14] Scherler, A., Jacquier, N., Kebbi-Beghdadi, C. & Greub, G. Diverse stress-inducing treatments cause distinct aberrant body morphologies in the Chlamydia-related bacterium, *Waddlia chondrophila*. *Microorganisms* **8**, 89 (2020). 31936490 10.3390/microorganisms8010089 PMC7022761

[15] Jacquier, N., Frandi, A., Pillonel, T., Viollier, P. H. & Greub, G. Cell wall precursors are required to organize the chlamydial division septum. *Nat. Commun.* **5**, 3578 (2014). 24709914 10.1038/ncomms4578 PMC3988822

[16] Vanover, J. et al. *Herpes simplex virus* co-infection-induced *Chlamydia trachomatis* persistence is not mediated by any known persistence inducer or anti-chlamydial pathway. *Microbiology* **154**, 971–978 (2008). 18310043 10.1099/mic.0.2007/012161-0

[17] Pospischil, A., Borel, N., Chowdhury, E. H. & Guscetti, F. Aberrant chlamydial developmental forms in the gastrointestinal tract of pigs spontaneously and experimentally infected with *Chlamydia suis*. *Vet. Microbiol.* **135**, 147–156 (2009). 18950970 10.1016/j.vetmic.2008.09.035

[18] Phillips-Campbell, R., Kintner, J., Whittimore, J. & Schoborg, R. V. *Chlamydia muridarum* enters a viable but non-infectious state in amoxicillin-treated BALB/c mice. *Microbes Infect* **14**, 1177–1185 (2012). 22943883 10.1016/j.micinf.2012.07.017 PMC3654801

[19] Workowski, K. A., Lampe, M. F., Wong, K. G., Watts, M. B. & Stamm, W. E. Long-term eradication of *Chlamydia trachomatis* genital infection after antimicrobial therapy: Evidence against persistent infection. *JAMA* **270**, 2071–2075 (1993). 8305018

[20] Borel, N. et al. Evidence for persistent *Chlamydia pneumoniae* infection of human coronary atheromas. *Atherosclerosis* **199**, 154–161 (2008). 18028932 10.1016/j.atherosclerosis.2007.09.026

[21] Brockett, M. R. & Liechti, G. W. Persistence alters the interaction between chlamydia trachomatis and its host cell. *Infect. Immun.* **89**(8), e00685-e720 (2021). 34001559 10.1128/IAI.00685-20 PMC8281235

[22] Wang, M. et al. Impacts of penicillin G on *Chlamydia trachomatis* serovar D and host HeLa cells in genital infections. *Arch. Microbiol.* **207**, 164 (2025). 40448745 10.1007/s00203-025-04365-5

[23] Huang, Y. et al. Robust Heat shock response in chlamydia lacking a typical heat shock sigma factor. *Front. Microbiol.* **12**, 812448 (2022). 35046926 10.3389/fmicb.2021.812448 PMC8762339

[24] Pokorzynski, N. D., Hatch, N. D., Ouellette, S. P. & Carabeo, R. A. The iron-dependent repressor YtgR is a tryptophan-dependent attenuator of the trpRBA operon in *Chlamydia trachomatis*. *Nat. Commun.* **11**, 6430 (2020). 33353937 10.1038/s41467-020-20181-5 PMC7755916

[25] Pokorzynski, N. D., Alla, M. R. & Carabeo, R. A. Host cell amplification of nutritional stress contributes to persistence in *Chlamydia trachomatis*. *MBio* **13**, e02719-22 (2022). 36377897 10.1128/mbio.02719-22 PMC9765610

[26] Kozusnik, T., Adams, S. E. & Greub, G. Aberrant bodies: an alternative metabolic homeostasis allowing survivability?. *Microorganisms* **12**, 495 (2024). 38543546 10.3390/microorganisms12030495 PMC10972484

[27] Slade, J. A., Brockett, M., Singh, R., Liechti, G. W. & Maurelli, A. T. Fosmidomycin, an inhibitor of isoprenoid synthesis, induces persistence in *Chlamydia* by inhibiting peptidoglycan assembly. *PLoS Pathog.* **15**, e1008078 (2019). 31622442 10.1371/journal.ppat.1008078 PMC6818789

[28] Pokorzynski, N. D., Thompson, C. C. & Carabeo, R. A. Ironing out the Unconventional mechanisms of iron acquisition and gene regulation in chlamydia. *Front. Cell Infect. Microbiol.* **7**, 394 (2017). 28951853 10.3389/fcimb.2017.00394 PMC5599777

[29] Dill, B. D. & Raulston, J. E. Examination of an inducible expression system for limiting iron availability during *Chlamydia trachomatis* infection. *Microbes Infect.* **9**, 947–953 (2007). 17544798 10.1016/j.micinf.2007.03.017 PMC2083192

[30] Thompson, C. C. & Carabeo, R. A. An optimal method of iron starvation of the obligate intracellular pathogen, *Chlamydia trachomatis*. *Front. Microbiol.* **2**, 20 (2011). 21687412 10.3389/fmicb.2011.00020 PMC3109288

[31] Brinkworth, A. J., Wildung, M. R. & Carabeo, R. A. Genomewide transcriptional responses of iron-starved *Chlamydia trachomatis* reveal prioritization of metabolic precursor synthesis over protein translation. *mSystems* **3**, e00184-17 (2018). 29468197 10.1128/mSystems.00184-17 PMC5811630

[32] Rockey, D. D., Scidmore, M. A., Bannantine, J. P. & Brown, W. J. Proteins in the chlamydial inclusion membrane. *Microbes Infect.* **4**, 333–340 (2002). 11909744 10.1016/s1286-4579(02)01546-0

[33] Scidmore-Carlson, M. A., Shaw, E. I., Dooley, C. A., Fischer, E. R. & Hackstadt, T. Identification and characterization of a *Chlamydia trachomatis* early operon encoding four novel inclusion membrane proteins. *Mol. Microbiol.* **33**, 753–765 (1999). 10447885 10.1046/j.1365-2958.1999.01523.x

[34] Agaisse, H. & Derré, I. Expression of the effector protein IncD in *Chlamydia trachomatis* mediates recruitment of the lipid transfer protein CERT and the endoplasmic reticulum-resident protein VAPB to the inclusion membrane. *Infect. Immun.* **82**, 2037–2047 (2014). 24595143 10.1128/IAI.01530-14 PMC3993449

[35] Pha, K. et al. The *Chlamydia* effector IncE employs two short linear motifs to reprogram host vesicle trafficking. *Cell Rep.* **43**, 114624 (2024). 39154341 10.1016/j.celrep.2024.114624 PMC12108946

[36] Gauliard, E., Ouellette, S. P., Rueden, K. J. & Ladant, D. Characterization of interactions between inclusion membrane proteins from Chlamydia trachomatis. *Front Cell Infect Microbiol.* **5**, 13 (2015). 25717440 10.3389/fcimb.2015.00013 PMC4324299

[37] Verbeke, P. et al. Recruitment of BAD by the *Chlamydia trachomatis* vacuole correlates with host-cell survival. *PLoS Pathog.* **2**, e45 (2006). 16710454 10.1371/journal.ppat.0020045 PMC1463014

[38] Wolf, K. et al. Treatment of *Chlamydia trachomatis* with a small molecule inhibitor of the *Yersinia* type III secretion system disrupts progression of the chlamydial developmental cycle. *Mol. Microbiol.* **61**, 1543–1555 (2006). 16968227 10.1111/j.1365-2958.2006.05347.x PMC1615999

[39] Jacob-Dubuisson, F., Mechaly, A., Betton, J.-M. & Antoine, R. Structural insights into the signalling mechanisms of two-component systems. *Nat. Rev. Microbiol.* **16**, 585–593 (2018). 30008469 10.1038/s41579-018-0055-7

[40] Zschiedrich, C. P., Keidel, V. & Szurmant, H. Molecular mechanisms of two-component signal transduction. *J. Mol. Biol.* **428**, 3752–3775 (2016). 27519796 10.1016/j.jmb.2016.08.003 PMC5023499

[41] Stock, A. M., Robinson, V. L. & Goudreau, P. N. Two-component signal transduction. *Annu. Rev. Biochem.* **69**, 183–215 (2000). 10966457 10.1146/annurev.biochem.69.1.183

[42] Ardissone, S. et al. Transcriptional landscape of *Waddlia chondrophila* aberrant bodies induced by iron starvation. *Microorganisms* **8**, 1848 (2020). 33255276 10.3390/microorganisms8121848 PMC7760296

[43] Pokorzynski, N. D., Brinkworth, A. J. & Carabeo, R. A bipartite iron-dependent transcriptional regulation of the tryptophan salvage pathway in *Chlamydia trachomatis*. *Elife* **8**, e42295 (2019). 30938288 10.7554/eLife.42295 PMC6504234

[44] Kozusnik, T., Kebbi-Beghdadi, C., Ardissone, S., Adams, S. E. & Greub, G. A conserved Chlamydiota-specific type III secretion system effector linked to stress response. *Microbiology* **171**, 001545 (2025). 40293431 10.1099/mic.0.001545 PMC12038028

[45] Qi, M. et al. *Chlamydia trachomatis* secretion of an immunodominant hypothetical protein (CT795) into host cell cytoplasm. *J. Bacteriol.* **193**, 2498–2509 (2011). 21441519 10.1128/JB.01301-10 PMC3133157

[46] Liu, X., Afrane, M., Clemmer, D. E., Zhong, G. & Nelson, D. E. Identification of *Chlamydia trachomatis* outer membrane complex proteins by differential proteomics. *J. Bacteriol.* **192**, 2852–2860 (2010). 20348250 10.1128/JB.01628-09 PMC2876478

[47] Subtil, A. et al. A directed screen for chlamydial proteins secreted by a type III mechanism identifies a translocated protein and numerous other new candidates. *Mol. Microbiol.* **56**, 1636–1647 (2005). 15916612 10.1111/j.1365-2958.2005.04647.x

[48] Belland, R. J. et al. Transcriptome analysis of chlamydial growth during IFN-gamma-mediated persistence and reactivation. *Proc. Natl. Acad. Sci. U S A* **100**, 15971–15976 (2003). 14673075 10.1073/pnas.2535394100 PMC307677

[49] Hanson, B. R. & Tan, M. Transcriptional regulation of the Chlamydia heat shock stress response in an intracellular infection. *Mol. Microbiol.* **97**, 1158–1167 (2015). 26075961 10.1111/mmi.13093 PMC4813507

[50] Helmlinger, L., Arthofer, P., Cyran, N., Collingro, A. & Horn, M. The adaptation of chlamydiae to facultative host multicellularity. *Curr. Biol.* **35**(14), 3368–3380 (2025). 40602405 10.1016/j.cub.2025.06.014

[51] Pillonel, T., Tagini, F., Bertelli, C. & Greub, G. ChlamDB: A comparative genomics database of the phylum chlamydiae and other members of the planctomycetes-verrucomicrobiae-chlamydiae superphylum. *Nucleic Acids Res.* **48**, D526–D534 (2020). 31665454 10.1093/nar/gkz924 PMC7145651

[52] Soules, K. R., LaBrie, S. D., May, B. H. & Hefty, P. S. Sigma 54-regulated transcription is associated with membrane reorganization and type III secretion effectors during conversion to infectious forms of *Chlamydia trachomatis*. *MBio* **11**, e01725-20 (2020). 32900805 10.1128/mBio.01725-20 PMC7482065

[53] Mathews, S. A. & Timms, P. Identification and mapping of Sigma-54 promoters in*Chlamydia trachomatis*. *J. Bacteriol.* **182**, 6239–6242 (2000). 11029448 10.1128/jb.182.21.6239-6242.2000 PMC94762

[54] Hogan, R. J., Mathews, S. A., Mukhopadhyay, S., Summersgill, J. T. & Timms, P. Chlamydial Persistence: beyond the Biphasic Paradigm. *Infect. Immun.* **72**, 1843–1855 (2004). 15039303 10.1128/IAI.72.4.1843-1855.2004 PMC375192

[55] Choudhary, K. S. et al. Elucidation of regulatory modes for five two-component systems in *Escherichia coli* reveals novel relationships. *mSystems*10.1128/msystems.00980-20 (2020). 33172971 10.1128/mSystems.00980-20 PMC7657598

[56] Theodorou, M. C., Panagiotidis, C. A., Panagiotidis, C. H., Pantazaki, A. A. & Kyriakidis, D. A. Involvement of the AtoS-AtoC signal transduction system in poly-(R)-3-hydroxybutyrate biosynthesis in Escherichia coli. *Biochim. Biophys. Acta* **1760**, 896–906 (2006). 16564134 10.1016/j.bbagen.2006.01.020

[57] Oshima, T. et al. Transcriptome analysis of all two-component regulatory system mutants of Escherichia coli K-12. *Mol. Microbiol.* **46**, 281–291 (2002). 12366850 10.1046/j.1365-2958.2002.03170.x

[58] Theodorou, M. C., Theodorou, E. C. & Kyriakidis, D. A. Involvement of AtoSC two-component system in Escherichia coli flagellar regulon. *Amino Acids* **43**, 833–844 (2012). 22083893 10.1007/s00726-011-1140-7

[59] Koo, I. C. & Stephens, R. S. A Developmentally Regulated Two-component Signal Transduction System in *Chlamydia**. *J. Biol. Chem.* **278**, 17314–17319 (2003). 12600998 10.1074/jbc.M212170200

[60] Koo, I. C., Walthers, D., Hefty, P. S., Kenney, L. J. & Stephens, R. S. ChxR is a transcriptional activator in Chlamydia. *Proc. Natl. Acad. Sci. U. S. A.* **103**, 750–755 (2006). 16407127 10.1073/pnas.0509690103 PMC1325966

[61] Hatch, N. D. & Ouellette, S. P. Identification of the alternative sigma factor regulons of *Chlamydia trachomatis* using multiplexed CRISPR interference. *mSphere* **8**, e00391-23 (2023). 37747235 10.1128/msphere.00391-23 PMC10597470

[62] Barta, M. L. et al. Atypical response regulator ChxR from chlamydia trachomatis is structurally poised for DNA binding. *PLoS ONE* **9**, e91760 (2014). 24646934 10.1371/journal.pone.0091760 PMC3960148

[63] Sharma, D., Garg, A., Kumar, M., Rashid, F. & Khan, A. U. Down-regulation of flagellar, fimbriae, and pili proteins in carbapenem-resistant *Klebsiella pneumoniae* (NDM-4) clinical isolates: a novel linkage to drug resistance. *Front. Microbiol.*10.3389/fmicb.2019.02865 (2019). 31921045 10.3389/fmicb.2019.02865 PMC6928051

[64] Lippa, A. M. & Goulian, M. Feedback inhibition in the PhoQ/PhoP signaling system by a membrane Peptide. *PLoS Genet.* **5**, e1000788 (2009). 20041203 10.1371/journal.pgen.1000788 PMC2789325

[65] Yang, C. et al. *Chlamydia trachomatis* ChxR is a transcriptional regulator of virulence factors that function in in vivo host–pathogen interactions. *Pathog. Dis.* **75**, ftx035 (2017). 28369275 10.1093/femspd/ftx035 PMC5974934

[66] Foschi, C. et al. Insights into penicillin-induced *Chlamydia trachomatis* persistence. *Microb. Pathog.* **142**, 104035 (2020). 32017957 10.1016/j.micpath.2020.104035

[67] Huston, W. M., Theodoropoulos, C., Mathews, S. A. & Timms, P. Chlamydia trachomatis responds to heat shock, penicillin induced persistence, and IFN-gamma persistence by altering levels of the extracytoplasmic stress response protease HtrA. *BMC Microbiol.* **8**, 190 (2008). 18986550 10.1186/1471-2180-8-190 PMC2585093

[68] Pokorzynski, N. D., Alla, M. R. & Carabeo, R. A. Host cell amplification of nutritional stress contributes to persistence in chlamydia trachomatis. *MBio* **13**(6), e02719-e2722 (2022). 36377897 10.1128/mbio.02719-22 PMC9765610

[69] Riffaud-Widner, C. M., Widner, R. E., Ouellette, S. P. & Rucks, E. A. Effect of tryptophan starvation on inclusion membrane composition and chlamydial-host interactions. 2024.11.26.625498 Preprint at 10.1101/2024.11.26.625498 (2024). 10.1128/iai.00532-24 PMC11834466 39804088

[70] Vollmuth, N. et al. c-Myc plays a key role in IFN-γ-induced persistence of Chlamydia trachomatis. *Elife* **26**(11), e76721 (2022). 10.7554/eLife.76721 PMC9512400 36155135

[71] Wu, C.-A., Chao, Y., Shiah, S.-G. & Lin, W.-W. Nutrient deprivation induces the Warburg effect through ROS/AMPK-dependent activation of pyruvate dehydrogenase kinase. *Biochimica et Biophysica Acta (BBA) – Mol. Cell Res.* **1833**, 1147–1156 (2013). 10.1016/j.bbamcr.2013.01.025 23376776

[72] Kassis, S., Grondin, M. & Averill-Bates, D. A. Heat shock increases levels of reactive oxygen species, autophagy and apoptosis. *Biochimica et Biophysica Acta (BBA) – Mol. Cell Res.* **1868**, 118924 (2021). 10.1016/j.bbamcr.2020.118924 33301820

[73] Bolger, A. M., Lohse, M. & Usadel, B. Trimmomatic: a flexible trimmer for Illumina sequence data. *Bioinformatics* **30**, 2114–2120 (2014). 24695404 10.1093/bioinformatics/btu170 PMC4103590

[74] Li, H. Aligning sequence reads, clone sequences and assembly contigs with BWA-MEM. Preprint at 10.48550/arXiv.1303.3997 (2013).

[75] Robinson, M. D., McCarthy, D. J. & Smyth, G. K. edgeR: A Bioconductor package for differential expression analysis of digital gene expression data. *Bioinformatics* **26**, 139–140 (2010). 19910308 10.1093/bioinformatics/btp616 PMC2796818

[76] Robinson, M. D. & Oshlack, A. A scaling normalization method for differential expression analysis of RNA-seq data. *Genome Biol.* **11**, R25 (2010). 20196867 10.1186/gb-2010-11-3-r25 PMC2864565

[77] Kolde, R. pheatmap: Pretty Heatmaps. (2019).

[78] Kanehisa, M., Furumichi, M., Tanabe, M., Sato, Y. & Morishima, K. KEGG: new perspectives on genomes, pathways, diseases and drugs. *Nucleic Acids Res.* **45**, D353–D361 (2017). 27899662 10.1093/nar/gkw1092 PMC5210567

