# Supplementary material for: Iron limitation-induced modulation of transcription in Chlamydia trachomatis
Source: Sci Rep. 2026 Apr 27;16:19479. doi: 10.1038/s41598-026-48043-y (PMC13287674; doi:10.1038/s41598-026-48043-y)
Supplement: Supplementary file 1 — Supplementary Information 1 [file 41598_2026_48043_MOESM1_ESM.docx]

**Supplementary data**


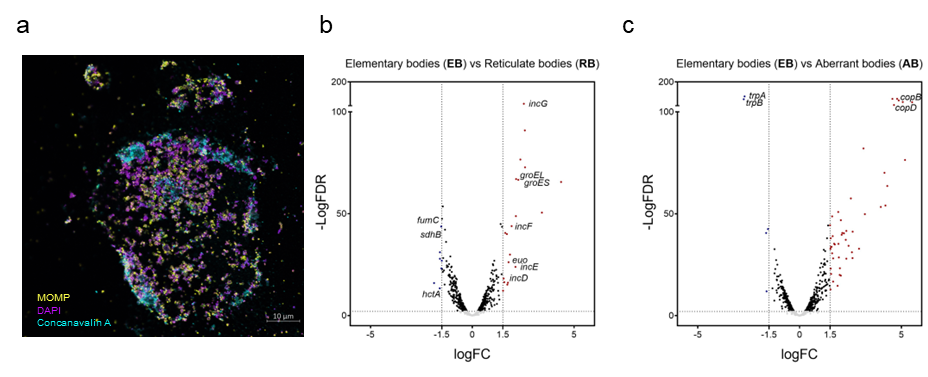


**Supplementary Figure 1. Confocal imaging and differential gene expression of *C. trachomatis* developmental stages.** (a) Confocal microscopy of C. trachomatis infection in HEp-2 cells at 72 hpi, showing extracellular EBs released by host cell disruption. Cells were fixed and labeled with Concanavalin A (turquoise), DAPI (purple), and anti-MOMP antibodies (yellow). Scale bars: 10 µm. (b–c) Volcano plots showing differential gene expression between *C. trachomatis* developmental stages: (b) EBs vs RBs and (c) EBs vs ABs. Each point represents the average expression of one gene from three replicates. Colored points indicate significantly differentially expressed genes with FDR ≤ 0.01 and log₂FC ≤ −1.5 (blue) or ≥ 1.5 (red).

**
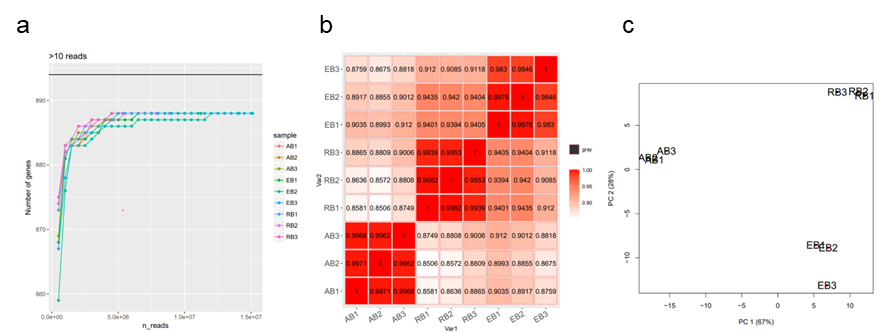
**

**Supplementary Figure 2. Variability in gene expression and sequencing depth analysis**

(a) Rarefaction analysis showing the number of genes mapped by at least 10 reads across all RNA sequencing samples. The curves illustrate the sequencing depth and gene discovery rate for each condition. Triplicates were used for each condition, ensuring reproducibility and consistency in gene expression analysis. (b) Pearson correlation matrix of log₂-transformed read counts. (c) Principal component analysis (PCA) of gene expression (first two principal components). All replicates showed low within-group variability, although the EB3 sample exhibited a slightly wider spread, indicating low variability in gene expression. Inter-group variability was high.


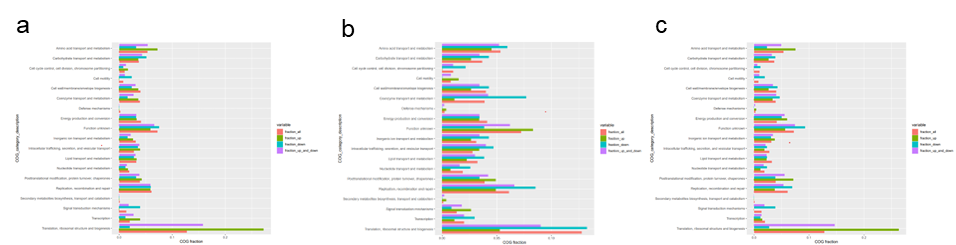


**Supplementary Figure 3. COG functional categorization of differentially expressed genes.**

(a) EBs vs RBs, (c) ABs vs RBs, and (c) EBs vs ABs. Genes were assigned to functional categories based on Clusters of Orthologous Groups (COG) annotations. Bar plots represent the fraction of genes in each category for the total differentially expressed genes (red), upregulated genes (green), downregulated genes (blue), and genes that were both up- and downregulated across comparisons (purple).

**Supplementary Table 1. Genes showing the highest change in expression between EBs vs RBs**

The table includes only the genes with a log_2_FC ≤ −1,5 or ≥1,5 and an FDR ≤ 0.01.

**Supplementary Table 2. Genes showing the highest change in expression between ABs vs RBs**

The table includes only the genes with a log_2_FC ≤ −1,5 or ≥1,5 and an FDR ≤ 0.01.

**Supplementary Table 3. Genes showing the highest change in expression between ABs vs EBs**

The table includes only the genes with a log_2_FC ≤ −1,5 or ≥1,5 and an FDR ≤ 0.01.

**Primers list**

***atoS***  200nM Fw CACATCCTCTGATCGCCGAA

Rv GATCGAGGGGAGTGCTTGTG

***atoC*** 300nM Fw CGAGGAGCACGTTAGACAGG

Rv GTACATCCGCTCTTCCTCGC

***chxR*** 250nM Fw ACATTCCTTGCTACAATCGCTT

Rv TTCGCTACATTCTGTTCCGGA

***incD*** 300nM Fw TCTAGTTGTTGCGGGCTTGT

Rv ACCGCTCTCTAGTCACAGCT

***incE*** 300nM Fw CAACTTGACGGATCCTGTGC

Rv AGAGCAAATCCACCACACGA

***incF*** 300nM Fw CTCTCGCCGCTCTTGTTTTG

Rv GTAGAAGCGATCCATCCCCG

***incG*** 300nM Fw TCCATATTATCAGCGGCGGT

Rv ACGAGCAGCCCCTTCAATTT

***trpR*** 300nM Fw CTGGCAAGCTTTTCTGACATT

Rv TTTGTGCGATACTGACTCCGT

***trpA*** 250nM Fw GCTGGTGATGGCGGTACTAG

Rv AGCTCGGATACCTTCTACGA

***trpB*** 250nM Fw TCAGGAAAACATGCAGCACG

Rv CCTGACTCATGCATTTCGGC

***htrA*** 250nM Fw AGGATTTTCTCGGGTCGCTG

Rv GCAATAGCCTGGTTCCCTGT

***groES*** 250nM Fw TGGCAGTGTCAGGAAGAATG

Rv CAAGCAACGACCCTCAAGAT

***wcw_0128*** 200nM Fw TTTGGGGAAAAGGGCTTGGT

Rv GGACAAAGCGGGCTAGATGT

***wcw_0985*** 200nM Fw TGCTCAAAAGGCTTGGCTG

Rv GGCAGTGAGGGCGATAATGG

***wcw_1136*** 300nM Fw TGTTCGACGGCATGATCACA

Rv GTAAAAGCGGGGAGAACGGA

***wcw_1137*** 300nM Fw AATCCAACGCTTATGACGCC

Rv GGCTTCGATTGGTTTGCTGA

***wcw_1571*** 300nM Fw CAAACATCGCAATCACGCTT

Rv CAATGCTCCTTCTTCTCCCC

***wcw_1572*** 300nM Fw AGGAGAGTCAGGCACAGGAA

Rv TAGCTCCTGTAAAGGCCCCT

***wcw_1870*** 200nM Fw TGGCGTGCAAACTTCTAGGA

Rv TCGCTCCTGTGGCATCTTTT

***wcw_0479*** 250nM Fw AGAGGCAGGAGAAGCATTGG

Rv CCAAGCGTTCCAAGCTGTTG
